# Role of Echocardiography in the Diagnosis of Light Chain Amyloidosis: A Case Report and Review of Literature

**DOI:** 10.7759/cureus.11377

**Published:** 2020-11-08

**Authors:** Mohamad Omar Choukair, Ahmad Halawi, Amal Nehmeh, Hasan Kazma

**Affiliations:** 1 Department of Cardiovascular Medicine, Lebanese University Faculty of Medical Sciences, Beirut, LBN; 2 Department of Internal Medicine, Lebanese University Faculty of Medical Sciences, Beirut, LBN; 3 Department of Cardiovascular Medicine, Bahman University Hospital, Beirut, LBN

**Keywords:** amyloidosis, echocardiography, immunoglobulin light-chain amyloidosis, cardiac amyloidosis

## Abstract

Amyloid light-chain (AL) amyloidosis is a rare disease with a broad clinical presentation that depends on the affected organ. Cardiac amyloidosis, a rare entity, can present as an isolated form of AL amyloidosis. This isolated form is considered a challenging diagnosis due to its broad nonspecific clinical presentation. In this article, we report a case of an adult male who presented with shortness of breath and was found to have many specific features of cardiac amyloidosis on echocardiography. In absence of other organ involvement, the results of the echocardiography directed us toward the diagnosis of AL cardiac amyloidosis. In addition, we highlight the role of echocardiography in the diagnosis of cardiac amyloidosis.

## Introduction

Amyloid light-chain (AL) amyloidosis is the most common form of systemic amyloidosis and is usually associated with an underlying plasma cell disorder [1]. The clinical presentation is broad, depending on the organ affected, and includes congestive heart failure, liver disease, nephrotic syndrome, neuropathy, and others [2]. The defective protein in AL amyloidosis is an immunoglobulin (Ig) light chain or a fragment of it [3]. AL cardiac amyloidosis can occur as a part of the spectrum of AL amyloidosis or as an isolated organ dysfunction [3]. The prevalence of isolated cardiac amyloidosis is thought to around five percent [4]. Several advances during the past decade have had a substantial impact on the approach to diagnose, treat, and improve the prognosis of AL amyloidosis [4]. Recently many diagnostic tools are being included in the workup such as serum free light-chain assay, cardiac magnetic resonance imaging, echocardiography, and serologic cardiac biomarkers, however, Immunohistopathology remains the gold standard in making the diagnosis of this disease [5]. Therefore, findings on echocardiography in AL cardiac amyloidosis are considered valuable in the early detection of this disease.

In this article, we report the case of a male patient who presented with shortness of breath and was found to have echocardiography findings of cardiac amyloidosis. In the absence of any other organ involvement, these findings helped us in making the diagnosis of isolated AL cardiac amyloidosis. In addition, we revise the role of echocardiography as a diagnostic tool for cardiac amyloidosis.

## Case presentation

A 63-year-old male patient presented to the emergency department complaining about a sudden onset of shortness of breath which started around four hours before. His history goes back to a few months prior to presentation when he started to experience dyspnea upon exertion before developing to become at rest. He also reported mild dry cough that started one month prior to presentation. He denied any fever, chills, chest pain, sputum production, lightheadedness, palpitations, or recent trauma. No recent travel or prolonged immobilization was reported. He is currently a smoker with 35 pack-year and he does not consume alcohol or illicit drugs. His past medical history included arterial hypertension, chronic obstructive pulmonary disease, and benign prostatic hyperplasia. No relevant surgical or family history was noted. His home medications included the following: furosemide 40 mg daily, bisoprolol 2.5 mg daily, tamsulosin 0.4 mg daily, spironolactone 25 mg daily, and omeprazole 20 mg daily. Upon presentation, his vital signs were within normal range with a blood pressure of 120/80 mmHg and a heart rate of 75 beats per minute. Cardiac physical examination showed regular rhythm, normal S1-S2 with no added murmurs or gallops, and no jugular vein distention or lower limbs edema. Lung examination was also unremarkable. No other important findings were noted on physical examination.

**Figure 1:**
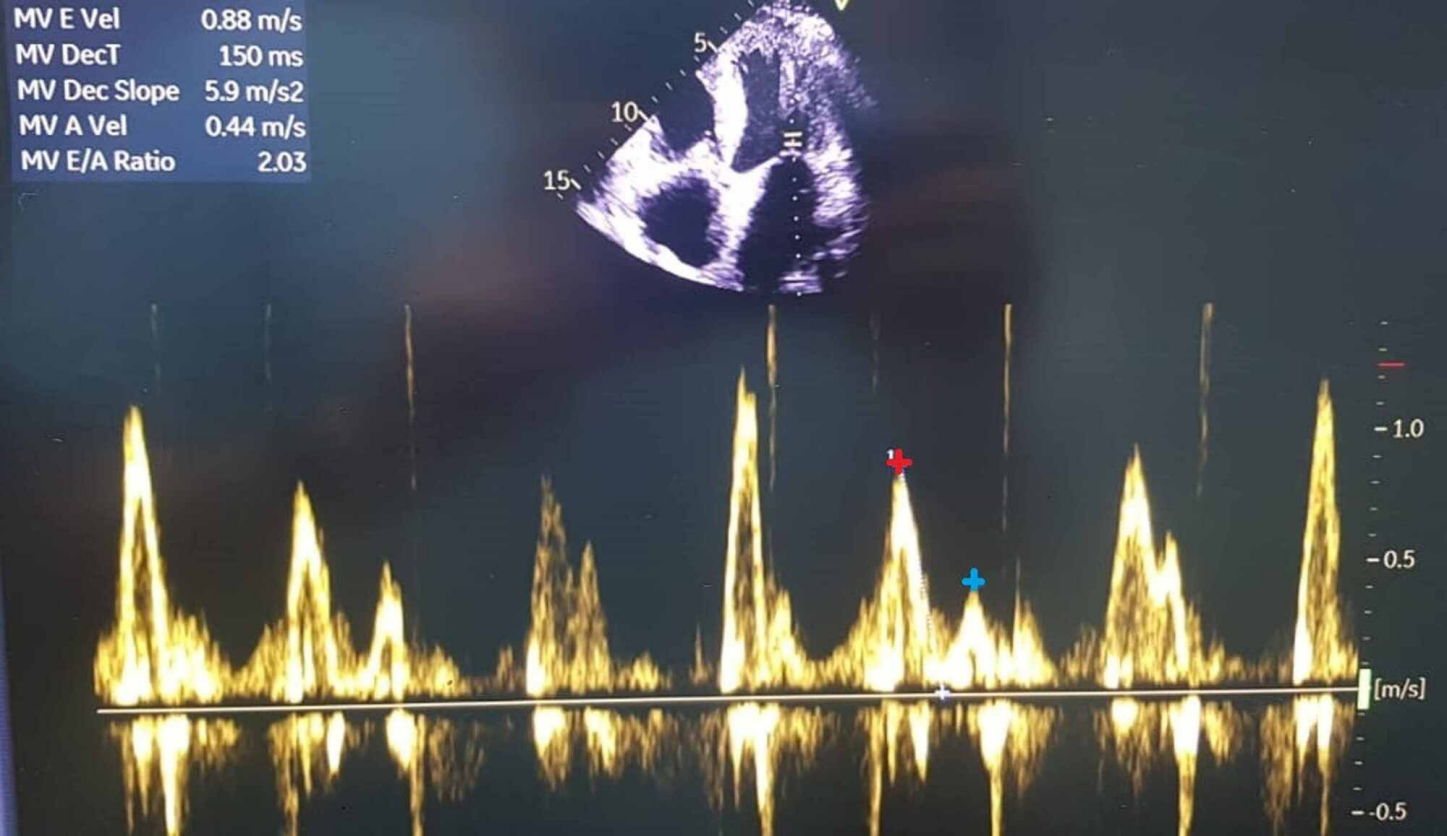
Four chamber view on pulse wave Doppler showing E/A: 2.03 Red + : E; Blue + : A

**Figure 2:**
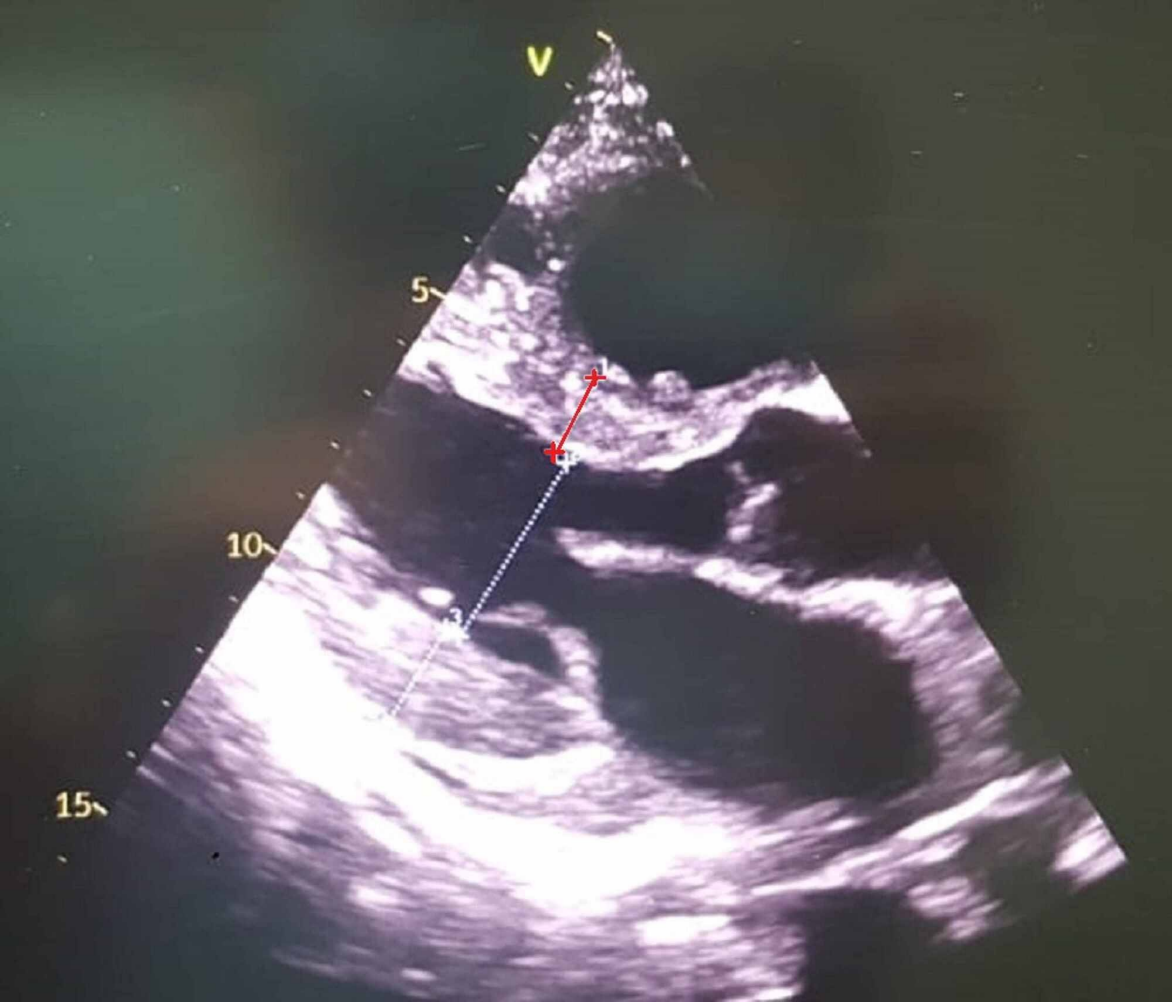
Parasternal long axis view echocardiography showing septal hypertrophy Red line: Septal hypertrophy

**Figure 3:**
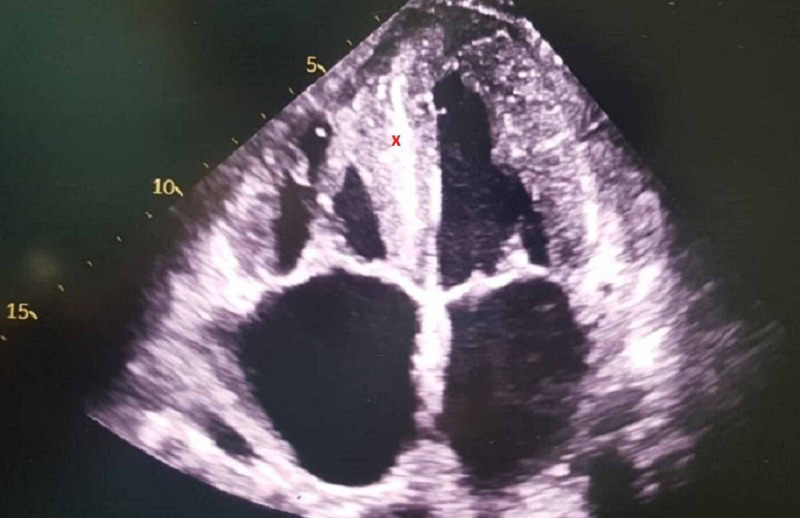
Four chamber view echocardiography showing septal hypertrophy x: Septal hypertrophy

**Figure 4:**
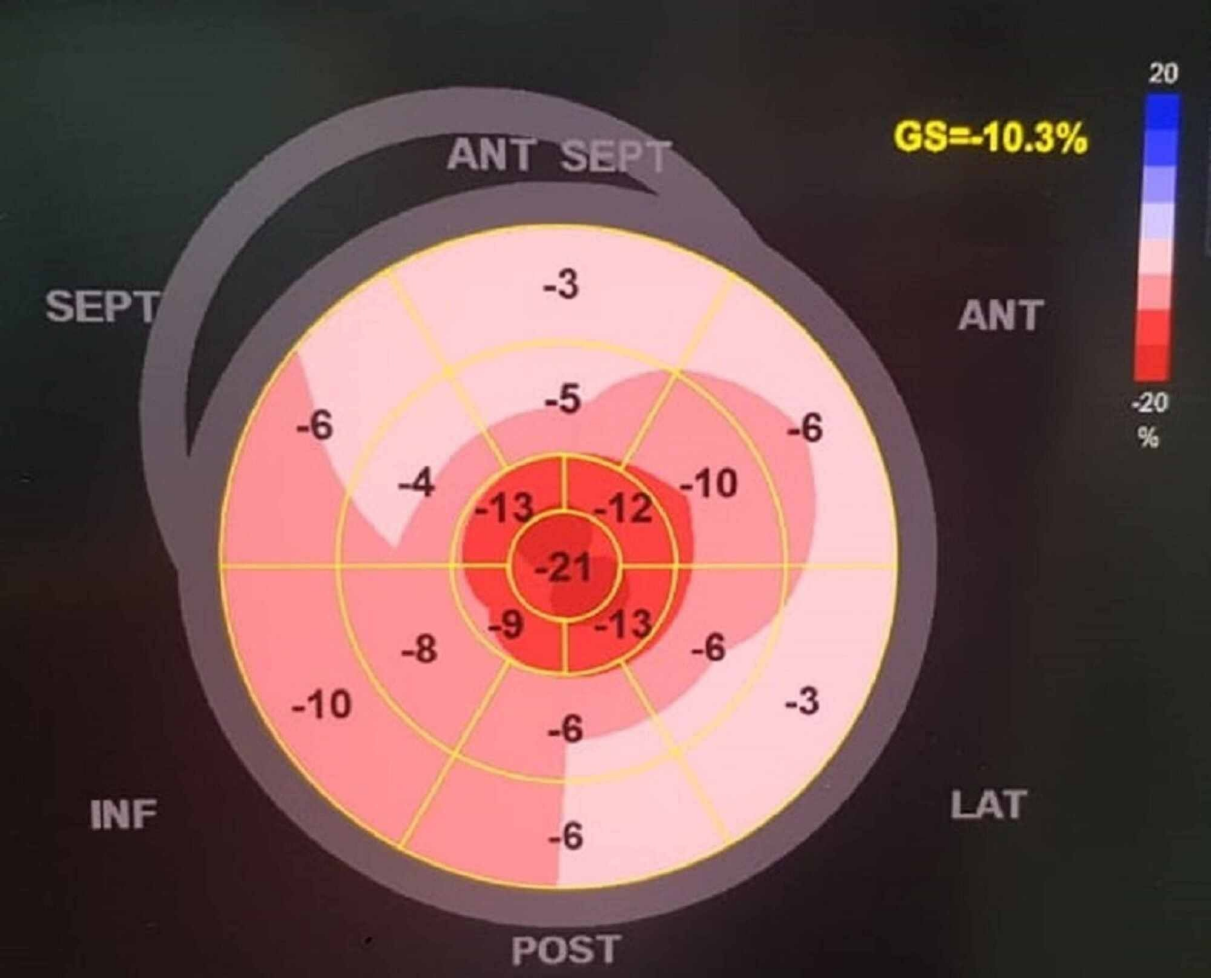
Apical sparing of the left ventricular longitudinal strain SEPT: Septal; ANT: Anterior; ANT SEPT: Anteroseptal; INF: Inferior; POST: Posterior; LAT: Lateral

Initial workup included normal laboratory findings of complete blood count and comprehensive metabolic panel. Electrocardiogram (ECG) showed low voltages in the limbs leads. And lastly, his chest radiograph showed cardiomegaly without any significant lung findings. In addition to these findings, echocardiography was performed in order to assess his cardiac function and to rule out heart failure as a possible cause for his dyspnea. The latter revealed moderate concentric hypertrophy with mild global hypokinesia, a reduced left ventricular ejection fraction of 42%, and an elevated left ventricular filling pressure E/A of more than 2.03 (Figures 1-3). In addition, an apical sparing of the left ventricular longitudinal strain consistent with cardiac amyloidosis was noted. The average peak systolic longitudinal strain was 10% (Figure 4).

Due to the discordance between the degrees of left ventricular thickness on echocardiography and QRS voltage on ECG, and the presence of apical sparing on strain technique, cardiac amyloidosis was considered as a possible etiology for this cardiomyopathy. Following echocardiography results, serum protein electrophoresis was performed to evaluate for an underlying gammapathy. Results showed a monoclonal spike (M spike) in the gamma region. Serum free kappa to lambda light chain ratio was low with a predominance of monoclonal bands of IgG lambda type in the gamma region. Urine immunofixation was negative for immunoglobulin light chain. Finally, bone marrow biopsy was performed and revealed five percent mature plasma cells, expression of kappa and lambda chain, with positive amyloid A. Congo red stain showed amyloid deposit on histology, compatible with the diagnosis of amyloidosis. Our patient was treated with optimal heart failure medication and started on bortezomib 1.3 mg/m2 per dose for two weeks after which the patient's condition stabilized.

## Discussion

At least 30 low-molecular-weight proteins are capable of forming amyloid fibrils. When these fibrils are extensive, they can interfere with normal function of the diseased organ [6]. Our patient had primary systemic (AL) amyloidosis, which occurs when a monoclonal population of plasma cells generates excess immunoglobulin light chains. AL amyloidosis can affect many organs leading to different clinical manifestations, although one organ system can predominate [3]. Cardiac infiltration primarily leads to arrhythmias and/or restrictive cardiomyopathy and is the second most common presentation of AL amyloidosis after nephrotic syndrome [7]. The classic clinical presentation is related to signs and symptoms of congestive heart failure including a rapid progression from exertional dyspnea to dyspnea at rest and orthopnea [3]. Fatigue and weakness are also common, resulting from the compromised cardiac output. Symptoms of right ventricular failure, including abdominal distension and lower extremity edema, may be more prominent in certain patients [8]. Coronary vessel involvement may manifest with angina, while palpitations and syncope may be seen with various conduction abnormalities associated with this condition, with atrial fibrillation being the most commonly identified arrhythmia and is almost always present [3,8]. Diagnosing cardiac amyloidosis requires a high index of suspicion due to its relatively low incidence. An early diagnosis is important in improving the patient's outcomes [4]. Typically, a combination of laboratory tests, ECG, cardiac imaging, and histopathology is required.

ECG is usually a rapid, simple test that may sometimes suggest cardiac amyloidosis, and abnormal findings are seen in approximately 50%-60% of cases [4,8]. Hence, the absence of low QRS voltage does not rule out cardiac amyloidosis. The typical finding is low QRS voltages that does not reflect the presence of left ventricular thickness [9,10]. A pseudo-infarct pattern with Q waves in the early precordial leads could also be seen leading to unnecessary invasive cardiac intervention [9]. Other ECG findings include atrial fibrillation, various degrees of atrioventricular block, and other conduction problems [4]. In our patient, the ECG only showed low QRS without other findings.

The echocardiography findings in cardiac amyloidosis are nonspecific in the early stages of the disease but could be pathognomonic with disease progression [11]. The hallmark finding is symmetric biventricular wall thickening with a spectrum of diastolic abnormalities varying from abnormal relaxation to a restrictive filling pattern [5,12]. Cardiac amyloidosis should be considered with left ventricular wall thickness greater than 12 mm that is not explained by a previous history of hypertension [4]. Atrial function is commonly impaired in cardiac amyloidosis due to the combination of deposition of fibrils in the atria and elevated LV filling pressures [11]. Thickening of the heart valves is also a common finding, which was present in the patient presented above. This impairment helps to distinguish cardiac amyloidosis from hypertensive heart disease. The reduction in left ventricle ejection fraction is seen later in disease course [12]. An apical sparing pattern of strain on 2D speckle echocardiogram is considered specific for cardiac amyloidosis enabling differentiation from other hypertrophic cardiac diseases [12]. The pattern of strain is due to a decrease in longitudinal strain in the basal and mid-wall regions in comparison to the apex where the strain is relatively preserved [8].

If the above findings on echocardiography are present, work up for a monoclonal process to evaluate for AL is always indicated. First, serum protein electrophoresis, serum immunofixation, and free light chain assay are mandatory [4]. In the presence of plasma dyscrasia, biopsy becomes indicated [4]. A biopsy can be taken from the bone marrow, cardiac or non-cardiac tissues. However, in case of absence of plasma cell dyscrasia, a myocardial scintigraphy assessment helps in making the diagnosis [4]. A negative biopsy from an extra cardiac site does not rule out cardiac amyloidosis and an endomyocardial biopsy should be obtained in such cases [2]. This procedure is, however, limited to larger referral centers with advanced facilities. In our case, the key findings on ECG and echocardiography that were discussed above contributed to the establishment of the correct diagnosis.

## Conclusions

AL cardiac amyloidosis is considered rare. However, it can lead to devastating outcomes if not treated properly. An early diagnosis is crucial for appropriate management which helps improve patient outcomes. Echocardiography is a rapid, simple test that has specific findings suggestive of cardiac amyloidosis. Hence, echocardiography can be considered a valuable tool permitting the early detection of this disease.
